# PCN/BiOCl Polymer-Based Heterojunction with Rich Chlorine Defects for Photocatalytic Amine Oxidation

**DOI:** 10.3390/polym15204145

**Published:** 2023-10-19

**Authors:** Guichuan Xu, Zhuhan Wang, Yefeng Chen, Li Qin, Limei Zhou

**Affiliations:** Chemical Synthesis and Pollution Control Key Laboratory of Sichuan Province, China West Normal University, Nanchong 637002, China; xuguichuan2023@163.com (G.X.); 15760592910@163.com (Z.W.); 13890791350@163.com (Y.C.); cwnuqinli@163.com (L.Q.)

**Keywords:** chlorine vacancy, Z-scheme heterojunction, amine oxidation, polymer-based, photocatalysis

## Abstract

Porous carbon nitride/bismuth oxychloride (PCN/BiOCl-x) polymer-based heterojunction photocatalysts were successfully synthesized via a simple in situ hydrothermal method. A PCN/BiOCl heterojunction with rich chlorine defects is prepared by adjusting the chlorine content of the BiOCl unit in the heterojunction by changing the solvent. The as-prepared catalysts were characterized via BET, SEM, TEM, XRD, XPS and optical testing, and they were used for a photocatalytic amine oxidation reaction. The results indicated that the catalytic performance of the PCN/BiOCl heterojunction was significantly enhanced due to the rich chlorine vacancies in the samples. The enhanced catalytic activity may be attributed to the Z-scheme heterojunction, abundant chlorine defects and large specific surface area. At the same time, the catalyst circulation experiment shows that the PCN/BiOCl heterojunction has good circulation performance.

## 1. Introduction

As an essential intermediate in the synthesis of pharmaceuticals and fine chemicals, imines have garnered widespread attention from researchers [1]. The traditional way to synthesize imines is through the reaction of aldehydes with amines, which requires expensive dehydrating agents and Lewis acids as catalysts. These methods are not only costly and energy-intensive, but they also produce by-products that can be harmful to the environment [2]. Therefore, searching and developing cleaner and more economical alternatives, as well as milder reaction conditions, holds significant scientific importance and promising application prospects.

Photocatalysis technology is an emerging field that integrates photochemistry, catalysis and materials chemistry. Utilizing photocatalysis to selectively oxidize amines to imines is a green and promising strategy. The commonly used photocatalysts for amine oxidation reactions include BiOCl, CdS, Fe_2_O_3_ and so on. Among them, BiOCl generates a strong internal electrostatic field between the [Bi_2_O_2_] unit and two chlorine atoms due to its unique layered structure; thus, the e^−^-h^+^ separation efficiency is improved [3]. However, BiOCl can only exhibit photocatalytic activity in the ultraviolet region due to its wide band gap (~3.5 eV) [4,5]. Therefore, the photocatalytic activity of BiOCl in practical applications is still unsatisfactory. Various methods have been reported to improve the photocatalytic activity of BiOCl.

More and more researchers suggest that the introduction of defects is one of the important ways to enhance the photocatalytic activity of photocatalysts [6,7]. Defects not only play a role in e^−^-h^+^ separation but may also form defect energy levels between the conduction band (CB) and valence band (VB), thus changing the light absorption ability of photocatalytic materials [8]. Currently, researchers have made some progress in defect engineering. For example, Hu et al. adjusted the defect distribution using continuous annealing in an O_2_/H_2_ atmosphere to obtain TiO_2_ photocatalysts with appropriate oxygen defects and higher charge carrier mobility [9]. The BiOCl material synthesized by Zeng et al. has oxygen defects on the surface, which can accelerate the capture and transfer of localized electrons, excite O_2_ into ·O_2_^−^ and promote the oxidation of photocatalytic NO [10]. Therefore, improving the photocatalytic performance of BiOCl by constructing defects has certain application prospects. 

**Figure 1:**
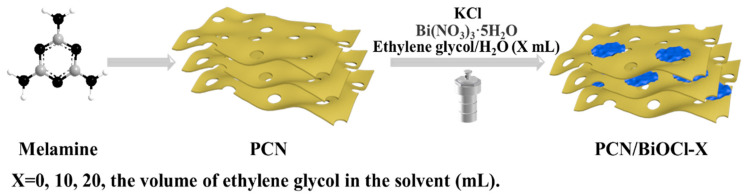
Simple synthesis diagram of PCN/BiOCl-X composite photocatalyst.

Graphitized carbon nitride (g-C_3_N_4_) is a layered polymer which has been widely used in the field of photocatalysis due to its low price, good stability and excellent optical properties [11]. In recent years, our research group synthesized a porous layered carbon nitride (PCN) by controlling the gas flow and applied it to the preparation of heterojunction photocatalysts [12]. In this work, we synthesized a high-surface-area Z-type heterojunction photocatalyst (PCN/BiOCl-X, where X represents the volume of ethylene glycol used in the preparation process) rich in chlorine defects via a simple solvothermal method (Figure 1). We investigated the photocatalytic activity of the as-prepared catalysts in the oxidative coupling reaction of benzylamine and the oxidative dehydrogenation reaction of secondary amines, and we studied their photocatalytic mechanism through various characterization methods. Compared with PCN, PCN/BiOCl-0 showed higher photocatalytic activity due to the following reasons: (1) PCN/BiOCl-0 exposes more active sites due to the larger specific surface area; (2) chlorine defects enhance its light absorption capacity; (3) the Z-type heterojunction accelerates the migration of charge carriers. In a word, this work provides a more promising method for the synthesis of imines.

## 2. Experimental Section 

### 2.1. Instruments and Chemicals

The elements and their distribution of the as-prepared samples were characterized via a scanning electron microscope (SEM, ZEISS, Sigma 500, Oberkochen, Germany) with energy-dispersive X-ray spectroscopy (EDS). The microstructures of the samples were characterized using a transmission electron microscope (TEM, JEOL, JEM-2100F, Showima City, Japan) with lattice fringe analysis to identify exposed crystal planes. The samples were also analyzed using an X-ray diffractometer (XRD, DONGNENG, TD3500, Shanghai, China) and compared to standard XRD cards to determine their phases. An infrared spectrometer (IR, Thermo Fisher Scientific, Nicolet-6700, Waltham, MA, USA) was applied to obtain the infrared spectrum for an analysis of the samples’ structures and chemical bonds. X-ray photoelectron spectroscopy (XPS, Thermo Fisher Scientific, ESCALAB 250Xi, Waltham, MA, USA) was used to analyze the chemical state and elemental composition of the samples. The UV-Vis spectrum (UV-Vis, SHIMADZU, UV-2600, Kiya-cho Nijyanan, Japan) and a fluorescence spectrometer (PL, Agilent, G9800A, Beijing, China) were used to analyze the optical properties of the as-prepared samples. The samples’ N_2_ adsorption–desorption isotherms were obtained using a fully automated physical adsorption instrument (Micromeritics, Mike-ASAP2020, Norcross, GA, USA) to analyze their specific surface areas (S_BET_) and pore size distributions. The active species involved in the photocatalytic reaction (including ·O_2_^−^, h^+^ and ·OH) were detected via the electron spin resonance spectrum (ESR, Bruker, A300, Billerica, MA, USA). The photoelectric properties of the samples were analyzed using an electrochemical workstation (Chenhua Instrument, CHI-660D, Shanghai, China). The wavelength of the xenon lamp (Perfect light, PLS-SXE 300+, Beijing, China) was 320–780 nm.

### 2.2. Sample Preparation

#### 2.2.1. Materials

Melamine (Adamas-beta, 99%, Shanghai, China), Bi(NO_3_)_3_·5H_2_O (Aladdin, AR, Shanghai, China), potassium chloride (Fuchen, AR, Tianjin, China), polyvinylpyrrolidone (Adamas-beta, k30, Shanghai, China), ethylene glycol (Keshi, GR, Chengdu, China), benzylamine (Aladdin, AR, Shanghai, China), acetonitrile (General, AR, Shanghai, China), dibenzylamine (Aladdin, AR, Shanghai, China), isopropanol (Keshi, AR, Chengdu, China), silver nitrate (Keshi, AR, Chengdu, China), p-benzoquinone (Heowns, 98%, Tianjin, China).

#### 2.2.2. Synthesis of PCN

Melamine (5 g) was heated to 300 °C under specific conditions (N_2_ flow and a heating rate of 5 °C·min^−1^). Then, the N_2_ flow was immediately stopped, and the temperature was further increased to 550 °C and maintained for 4 h. The collected product is named PCN. The PCN refers to porous carbon nitride; the PCN is porous because the porosity is relative to the bulk g-C_3_N_4_ (approximately 5–10 m^2^/g).

#### 2.2.3. Synthesis of PCN/BiOCl

Bismuth nitrate pentahydrate (Bi(NO_3_)_3_·5 H_2_O, 0.112 g), potassium chloride (KCl, 0.08 g) and polyvinylpyrrolidone (PVP, 0.10 g) were added to deionized water (20 mL). After stirring (430 rpm) for 0.5 h, PCN (0.3 g) was added and stirred for 40 min under room temperature. The mixture was then transferred to a hydrothermal reactor (100 mL); the oven temperature was raised from 25 °C to 180 °C after about 25 min and maintained for 24 h. Finally, the product was washed with water, centrifuged to remove the supernatant and then washed with ethanol, and the process was repeated three times. The product was vacuum-dried at 60 °C. By changing the solvent ratio (such as 20 mL ethylene glycol, 10 mL water and 10 mL ethylene glycol, 20 mL water, respectively), samples with different chlorine defects were prepared using the same method as above. The samples were named PCN/BiOCl-0, PCN/BiOCl-10 and PCN/BiOCl-20 according to the solvent ratio of ethylene glycol. The BiOCl monomer was prepared via the same synthesis method using water as the solvent without PCN. Without KCl, the PCN/Bi_2_O_3_ was prepared using the same synthesis method as PCN/BiOCl-0.

### 2.3. Photocatalytic Experiments

#### 2.3.1. Oxidation Coupling Reaction of Benzylamine

For the specific template reaction, 0.01 g of photocatalyst, 0.30 mmol of benzylamine and 10 mL of acetonitrile were added to a quartz photoreactor (25 mL). The quartz reactor is a double-layered glass instrument, where the reaction occurs in the inner layer and the water (room temperature) flows through the outer layer to cool the quartz reactor. 

To achieve adsorption–desorption equilibrium and ensure uniform mixing of the solution, the mixed solution was stirred in a dark environment for 0.5 h before being transferred to a xenon lamp for the photoreaction. In the reaction process, the problem of solvent evaporation nearly did not occur. After the reaction, the supernatant liquid was separated via centrifugation and analyzed using a gas chromatograph (GC, Agilent, 7890A, Beijing, China). 

#### 2.3.2. Dehydrogenation of Secondary Amines

Specifically, 0.01 g of photocatalyst, 0.15 mmol of dibenzylamine and 10 mL of acetonitrile were added to a 25 mL photoreactor. In order to reach adsorption–desorption equilibrium, the mixed solution was stirred in the dark for 0.5 h. The photoreaction was then carried out under xenon lamp irradiation. After the reaction, the supernatant liquid was separated via centrifugation and analyzed using a GC.

#### 2.3.3. Stability Testing

This is an experimental procedure for stability testing, which aims to evaluate the chemical stability of photocatalysts through a cycling test. Specifically, a certain amount of photocatalyst and substrate (amine) were dispersed in 10 mL of acetonitrile, and the mixture was allowed to equilibrate through dark reactions in a 25 mL photoreactor for 0.5 h. Then, the mixture was subjected to a photochemical reaction. All mixed solutions were centrifuged after the reaction, and the supernatant liquid (1 mL) was taken to determine the conversion rate and selectivity of the reaction via GC analysis. The photocatalyst was then recovered, washed with acetonitrile until there were no residues of the substrate (amine) or its products and finally dried at 60 °C. The collected catalyst was further subjected to photochemical reactions using the same procedure for multiple cycles to test its stability.

#### 2.3.4. Trapping Experiment

The trapping experiment was carried out to investigate the mechanism of the photocatalytic amine oxidation reaction. Specifically, the capture experiment is similar to the photocatalytic amine oxidation reaction, but excess capture agents (1 mM) need to be added to the reaction solution to detect the role of various active species (·O_2_^−^, ·OH, h^+^ and e^−^). In addition, in order to eliminate all active species generated by O_2_, the reaction was carried out in the N_2_ atmosphere.

## 3. Results and Discussion

### 3.1. Structure and Morphological Analysis

**Figure 2:**
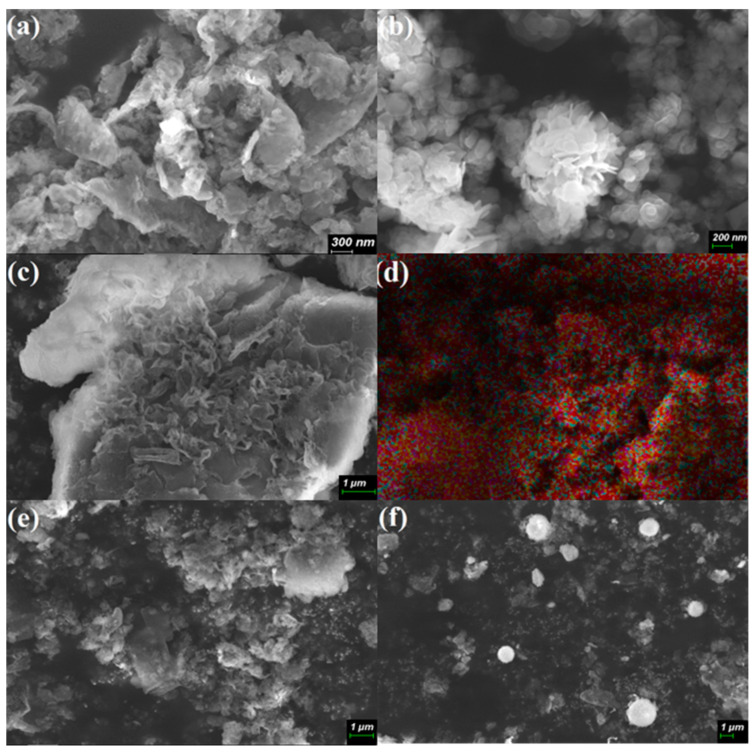
SEM images of PCN (**a**), BiOCl (**b**), PCN/BiOCl-0 (**c**); elemental mapping of PCN/BiOCl-0 (**d**), PCN/BiOCl-10 (**e**) and PCN/BiOCl-20 (**f**).

The morphology, elemental composition and distribution of the prepared PCN/BiOCl-0 composite photocatalyst were characterized via SEM (Figure 2). As shown in Figure 2a,b, both PCN and BiOCl monomers have a loose and porous layered structure. PCN/BiOCl-0 (Figure 2c) exhibits a layered structure, with small nano-sized BiOCl sheets loaded on top of large PCN sheets. Figure 2d is the elemental distribution map of PCN/BiOCl-0, which further proves that BiOCl is uniformly dispersed on PCN. PCN/BiOCl-10 (Figure 2e) also shows a stacked layer structure, but here the nano-sized BiOCl sheets tend to aggregate. The BiOCl in PCN/BiOCl-20 (Figure 2f) presents a nano-flower ball structure. The EDS was used to detect and analyze the elemental distribution of the samples, and their molar ratio was calculated according to the mass fraction of Bi and Cl displayed in Table S1. The molar ratio of Bi and Cl in both the monomeric BiOCl and all PCN/BiOCl composite materials is greater than the molar ratio of chlorine and bismuth elements in standard bismuth oxychloride, indicating the presence of chlorine defects in these photocatalytic materials. Among them, the value of the molar ratio of Bi and Cl in the PCN/BiOCl-0 composite material is the highest (5.71), indicating that PCN/BiOCl-0 has the most chlorine defects. According to the literature, the improved activity of the photocatalyst is due to the presence of defects that enhance the light absorption capacity of the photocatalyst [13].

**Figure 3:**
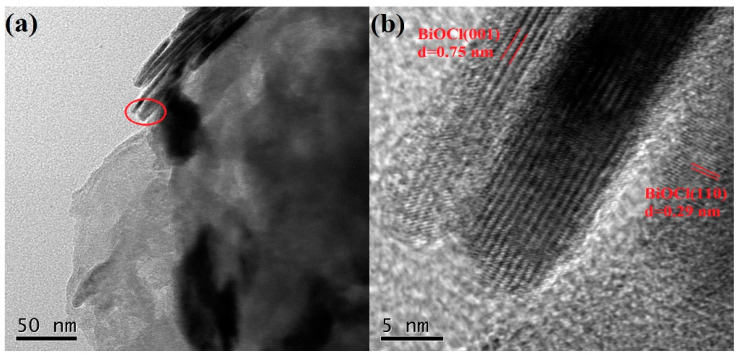
TEM (**a**) and HRTEM (**b**) images of PCN/BiOCl-0.

To further investigate the microstructure of the PCN/BiOCl-0 photocatalyst, TEM characterization of PCN/BiOCl-0 was performed (Figure 3). As shown in Figure 3a, small nanoscale BiOCl sheets (in the red cycle) were observed to be dispersed on large PCN layers. In the HRTEM image at 5 nm (Figure 3b), the clear lattice fringes demonstrate the good crystallinity of the photocatalytic material. The spacing of the lattice fringes was determined to be 0.75 nm and 0.29 nm, corresponding to the (001) and (110) crystal planes of BiOCl [14], which further confirms the successful loading of BiOCl onto the PCN surface.

**Figure 4:**
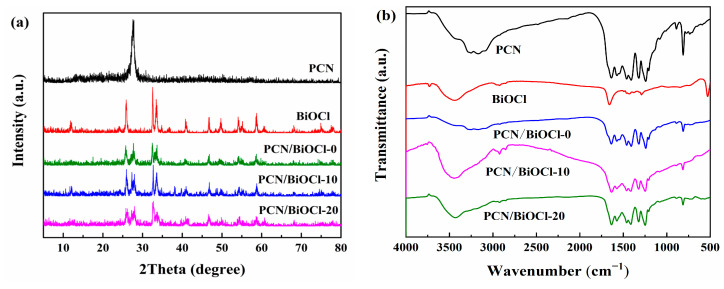
XRD curves (**a**) and infrared spectra of samples (**b**).

The crystal lattice configuration and phase composition of PCN/BiOCl-X were determined via XRD (Figure 4a). In the XRD pattern of pristine PCN, there are two diffraction peaks at around 13.8° (100) and 27.6° (002), respectively, which are attributed to the stacking of the structural units within the PCN plane and the interlayer spacing. The results are consistent with previous reports on nitrogen–carbon materials [15]. In the XRD pattern of pure BiOCl, multiple diffraction peaks were observed, corresponding to the peak positions of the standard card (JCPDS 06-0249), indicating the successful synthesis of BiOCl [16]. All PCN/BiOCl-X composites showed characteristic peaks of PCN and BiOCl, which confirmed the successful integration of these two materials, consistent with the TEM characterization results. Additionally, the infrared spectra of the prepared samples are shown in Figure 4b. The appearance of a peak at 814 cm^−1^ in the PCN monomer spectrum can be ascribed to the bending vibration of the 3-s-triazine ring [17]. The presence of peaks between 1190 and 1681 cm^−1^, including 1241, 1319, 1415, 1461 and 1636 cm^−1^, were characteristic of the stretching modes associated with the C-N=C heterocyclic ring in PCN [18]. The broad peak centered at 3237 cm^−1^ was assigned to the uncoupled amino group and the water molecule that was adsorbed on the surface [19]. Furthermore, the peak shifted to a lower wavenumber, which may be due to the hydrogen bonds between the hydroxyl and amino groups that may be present in the composite. In the IR spectrum of the BiOCl monomer, the peak at 529 cm^−1^ was associated with the Bi–O stretching vibrational mode [20,21]. The presence of the peak at 1654 cm^−1^ can be explained from the stretching of Cl–O bonds [22]. The PCN/BiOCl composite materials showed characteristic absorption peaks of PCN; however, the characteristic absorption peaks of BiOCl were not clear. The result was attributed to the low content of BiOCl in the samples and the strong overlap of the BiOCl peak with the PCN peak, making it difficult to distinguish. The specific surface area of the samples was detected using an automatic physical adsorption instrument and recorded in Table S2. The *S*_BET_ value of PCN/BiOCl-0 (65.86 m^2^·g^−1^) is the highest, indicating that the reaction’s active sites of PCN/BiOCl-0 are more fully exposed and conducive to come in contact with the substrate compared to those of PCN (30.04 m^2^·g^−1^), PCN/BiOCl-10 (58.97 m^2^·g^−1^) and PCN/BiOCl-20 (39.67 m^2^·g^−1^). The increase in the *S*_BET_ of PCN/BiOCl-0 may be due to two possible factors: (1) the BiOCl in the PCN/BiOCl-0 has a nanosheet structure, which has a larger *S*_BET_ than the flower ball-like BiOCl; (2) the presence of chlorine defects may lead to the formation of some pores [23].

### 3.2. Chemical State Analysis

**Figure 5:**
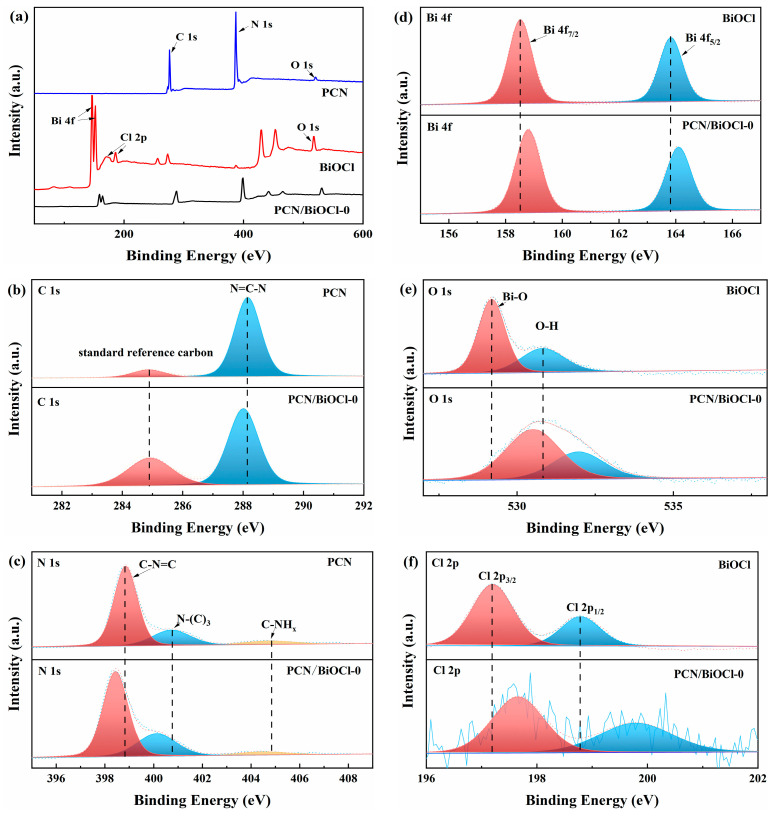
XPS survey spectra of different samples (**a**); XPS spectra of different samples (C, N, Bi, O, Cl) (**b**–**f**).

The surface electronic states and elemental compositions of the synthesized samples were analyzed via XPS (Figure 5). Figure 5a shows the full spectra of PCN, BiOCl and PCN/BiOCl-0, and all the signature peaks attributed to both PCN and BiOCl are observed in the PCN/BiOCl-0 composite material, indicating the successful synthesis of PCN/BiOCl-0 composite [15]. Figure 5b shows the high-resolution C 1s spectra of PCN and PCN/BiOCl-0. The PCN and PCN/BiOCl-0 have characteristic peaks at 284.8 eV and 288.1 eV, which are attributed to the standard reference carbon (the relative content of the standard reference carbon was not equal for different samples) and the N-C=N functional group in the aromatic ring with N, respectively [24,25]. Compared with monomeric PCN, the N-C=N functional group peak (278.98 eV) in the high-resolution C 1s spectrum of PCN/BiOCl-0 moves towards a lower binding energy. Both PCN and PCN/BiOCl-0 show three distinct peaks in N 1s high-resolution spectra (Figure 5c), which can be attributed to the sp^2^-hybridized C-N=C nitrogen, sp^3^-hybridized N-(C)_3_ nitrogen and the free amino nitrogen, indicating the presence of these chemical functionalities in both cases [26]. The N 1s high-resolution spectra of the PCN/BiOCl-0 composite material shows characteristic peaks at a lower binding energy than those of PCN, indicating the shift in the position of these peaks towards the left. As shown in Figure 5d, the high-resolution XPS spectrum of Bi 4f exhibits peaks at 158.4 and 163.8 eV, which originate from the Bi 4f_7/2_ and Bi 4f_5/2_ orbitals of BiOCl, respectively. These findings provide strong evidence for the existence of Bi^3+^ in the studied sample [27]. Compared with BiOCl, the high-resolution Bi 4f spectrum of the composite material moves towards a higher binding energy. Figure 5e shows the high-resolution O 1s spectra of BiOCl and PCN/BiOCl-0. According to the O 1s spectrum of BiOCl, two characteristic peaks situated at 529.2 and 530.8 eV can be ascribed to the Bi–O bond in BiOCl and surface-including water (-OH) species [28]. Figure S1 shows the O 1s spectra of PCN; the signal was very noisy due to the lower O elemental content, and the contribution of O to PCN/BiOCl can be negligible. Compared to pure BiOCl, the O 1s spectrum of PCN/BiOCl-0 shifts towards a higher binding energy. Figure 5f shows the high-resolution Cl 2p spectra, where BiOCl has characteristic peaks at 197.1 and 198.7 eV binding energy, corresponding to Cl 2p_3/2_ and Cl 2p_1/2_ of BiOCl, respectively, proving the existence of Cl in the material [29]. Compared with monomeric BiOCl, the peak strength of the Cl 2p signal of PCN/BiOCl-0 shifts towards a higher binding energy. In summary, after the formation of the complex material, a decrease in binding energy is observed in the C and N 1s spectra, while the Bi 4f, O 1s and Cl 2p spectra shift towards higher binding energies. The binding energy of elements tends to decrease as their electron density increases, and the electron density on the surface of BiOCl is lower than that on the surface of PCN. This result implies the occurrence of an electron transfer process from BiOCl to PCN, demonstrating the successful formation of a heterojunction structure between PCN and BiOCl [19]. Additionally, the Cl 2p spectrum of PCN/BiOCl-0 has poor signal-to-noise ratio and weak signal peak intensity, which is attributed to the low absolute content of chlorine, further proving the existence of chlorine defects in the composite.

### 3.3. Photoelectrochemical and Optical Properties

**Figure 6:**
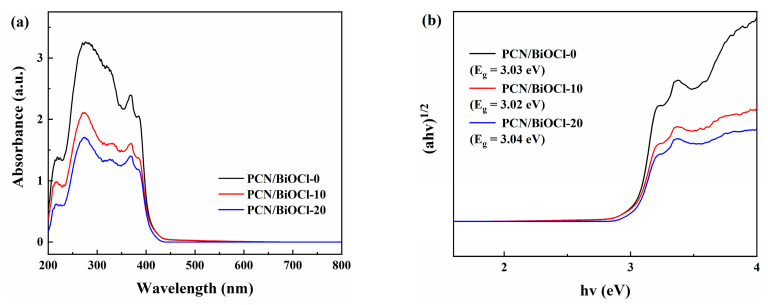
UV-Vis DRS (**a**) and band gap energy (**b**) of different samples.

The ability of the samples to absorb light was measured through UV-Vis, and the corresponding results are illustrated in Figure 6a. Compared to PCN/BiOCl-10 and PCN/BiOCl-20, PCN/BiOCl-0 has a higher light absorption intensity, which may be attributed to the chlorine defects in PCN/BiOCl-0 that cause the emergence of defect states between the conduction and valence bands of BiOCl. Previous studies indicated that the charge excitation from the defect states into the conduction band can improve the light absorption capability of the material [13]. The high light absorption capacity of PCN/BiOCl-0 is beneficial for promoting photocatalytic reactions. The absorption edge of PCN can be observed at 443 nm in Figure S2a. After loading with BiOCl, the absorption edge of PCN in the composite material undergoes blue shift (the absorption edge of BiOCl is located at 341 nm) (Figure S2b). The combination of PCN and BiOCl causes the absorption edge of the composite material to shift towards the midpoint of the two monomers, consistent with many previous studies [30]. The bandgap values of the photocatalysts were evaluated through the application of the Kubelka–Munk equation, and the bandgap values of PCN and BiOCl were 2.80 eV and 3.60 eV, respectively. Compared to PCN monomers, the bandgap values of PCN in all composite materials (Eg ≈ 3 eV) increased (Figure 6b), which may be due to the interaction between PCN and BiOCl, further confirming the successful combination of PCN and BiOCl.

**Figure 7:**
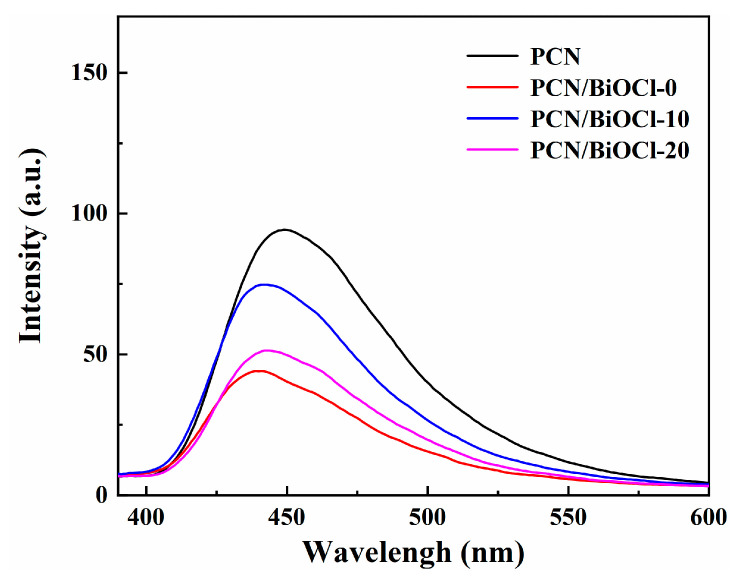
Fluorescence spectra of samples.

By measuring the fluorescence intensity, the rate of electron-hole recombination in the sample can be detected (Figure 7). Under illumination, part of the energy generated by the photo-induced electron-hole recombination in the photocatalyst is transferred to fluorescence [31]. The quenching of fluorescence indicates that the rate of electron-hole recombination is effectively suppressed. Thus, the weaker the PL emission peak of the photocatalyst, the higher rate of electron-hole recombination, indicating the photocatalytic performance was stronger. Figure 7 shows that the fluorescence emission peaks of the samples from high to low are PCN > PCN/BiOCl-10 > PCN/BiOCl-20 > PCN/BiOCl-0. Compared to the PCN monomer, the PL emission peak of the composite materials is weaker due to the formation of a heterojunction between PCN and BiOCl, which increases the efficiency of charge separation. The PL emission peak of PCN/BiOCl-0 is the weakest, indicating the best separation efficiency of its photogenerated charge carriers. 

The photocurrent response of the samples was studied using an electrochemical workstation. A stronger photocurrent response of a semiconductor photocatalyst indicates better charge separation efficiency [32]. Figure S3a shows the photocurrent of the samples under five intermittent light irradiations. The composite materials have a stronger photocurrent than the PCN and BiOCl monomers. Among them, the PCN/BiOCl-0 sample shows the strongest photocurrent response, implies that it has the highest mobility of photogenerated charge carriers and effectively suppresses the recombination of e–h^+^ pairs. This result is in line with the result of photoluminescence analysis.

The separation and recombination behavior of the photogenerated charge carriers was further investigated through EIS to analyze the conductivity of the samples under light and dark conditions. Figure S3b shows the impedance of the samples under illumination. The Nyquist plot of the samples’ arc radii from high to low is PCN > BiOCl > PCN/BiOCl-10 > PCN/BiOCl-20 > PCN/BiOCl-0. PCN/BiOCl-0 displays the smallest Nyquist plot arc radius, indicating the highest charge carrier mobility. Figure S3c shows the dark impedance of the samples, and the order of the arc radius size is the same as that of the light impedance. These results demonstrate that the charge carrier transport efficiency of PCN/BiOCl-0 composite material is superior, in agreement with the photocurrent testing results.

### 3.4. Photocatalytic Activity

The photocatalytic performance of the catalysts was evaluated through the photocatalytic coupling reaction of benzylamine, and the experimental results are listed in Table 1. All the catalysts showed excellent selectivity (~99.9%) for the photocatalytic coupling reaction of benzylamine. Compared with the monomer PCN (40.1%) and BiOCl (54.5%), the PCN/BiOCl-X composite materials showed significantly enhanced photocatalytic performance in the photocatalytic coupling reaction of benzylamine, which can be attributed to the formation of the heterojunction between PCN and BiOCl. The photocatalytic performance of the three composite materials was as follows: PCN/BiOCl-10 (72.7%) < PCN/BiOCl-20 (76.1%) < PCN/BiOCl-0 (88.0%) (Table 1, entries 3–5). Among them, PCN/BiOCl-0 had the highest photocatalytic activity, which may be attributed to two main factors: (1) a higher specific surface area allows the active sites on the catalyst to come in contact with substrate more completely; (2) the presence of chlorine defects makes the material have high light absorption intensity, which is beneficial for enhancing the photocatalytic performance. 

In order to explore the perfect conditions for the photocatalytic oxidation coupling reaction of phenylethylamine, optimization was carried out from two aspects, reaction time and catalyst dosage, and the experimental results are shown in Table 2. It was found that the PCN/BiOCl-0 photocatalyst had the best catalytic effect based on Table 1. Therefore, all subsequent reactions were catalyzed using PCN/BiOCl-0. Firstly, the optimization of the catalyst dosage was carried out (Table 2, entries 1–4). When the amount of the photocatalyst was 0 g, the conversion rate of benzylamine was only 2.7%, indicating that the photocatalyst was crucial in the reaction. Generally, when the catalyst dosage was increased, the conversion rate of the photocatalytic reaction also increased correspondingly. However, it can be observed from Table 2 that the amounts of the photocatalysts were 0.02 g and 0.03 g and the yields of the product were 68.3% and 66.2%, respectively. This result indicates that increasing the catalyst dosage does not significantly increase the yield of products. From the perspective of reaction results and economics, the optimal catalyst dosage was 0.02 g. 

When the catalyst dosage was 0.02 g, the optimization of the irradiation time was carried out (Table 2, entries 5–8). With the increase in irradiation time, the conversion rate of phenylethylamine also increased. When the irradiation time was 6 h, the conversion rate of phenylethylamine reached 97.2%. Under the condition without light (Table 2, entry 5), the conversion rate of the phenylethylamine was only 2.2%, which proves that light is an important condition for catalyzing the oxidation coupling reaction of benzylamine.

The scope of the reactants was expanded under the optimal reaction conditions, and the corresponding experimental results are documented in Table 3. The photocatalytic oxidation of amine derivatives gives rise to the formation of the corresponding imines, with impressive selectivity (~99%). Compared to electron-withdrawing groups, electron-donating groups were more reactive (Table 3, entries 2–4). By using the benzylamine derivatives substituted with a methyl group to study the steric hindrance effect, the conversion rates of the benzylamine derivatives were as follows: 4-methylbenzylamine (100%) > 3-methylbenzylamine (83.9%) > 2-methylbenzylamine (76.6%) (Table 3, entries 4–6). The results of the experiment indicated that the greater the steric hindrance of the reaction substrate, the more restricted the reaction proceeded. Furthermore, for the reaction of heterocyclic amines (Table 3, entries 7–9), good conversion rates (around 80–90%) were obtained within a given reaction time.

In order to broaden the application of the photocatalysts, a photocatalytic oxidation–dehydrogenation reaction was carried out on secondary amines. Using the oxidation–dehydrogenation of dibenzylamine as a template reaction, the photocatalytic activities of different catalysts were determined. The photocatalytic activity of the catalysts can be evaluated according to the conversion rate of dibenzylamine in Table 4, which suggests the following order: PCN (25.7%) < BiOCl (36.0%) < PCN/BiOCl-10 (39.9%) < PCN/BiOCl-20 (45.2%) < PCN/Bi_2_O_3_ (52.0%) < PCN/BiOCl-0 (59.1%). The PCN/BiOCl-0 exhibited the best photocatalytic activity, which may be attributed to the successfully constructed heterojunction structure, abundant chlorine defects and high specific surface area. This rule was consistent with the photocatalytic coupling reaction of benzylamine oxidation.

To further optimize the reaction conditions, the reaction was carried out with different amounts of catalyst and irradiation times. To be consistent with the coupling reaction of benzylamine, the amount of reactant was increased to 0.25 mmol. The different amounts of catalyst are compared in Table 5 (entries 1–4); the appropriate amount of catalyst was 0.02 g based on the conversion rate and selectivity of the photocatalytic oxidation–dehydrogenation of dibenzylamine. By comparing the reaction results at different irradiation times (Table 5, entries 5–9), when the irradiation time was increased from 3 h to 7 h, the conversion rate of dibenzylamine gradually increased, but the selectivity decreased. Side reactions may occur after a certain reaction time and lead to the formation of by-products. Taking all factors into account, the optimized irradiation time was 6 h.

A range of substrates were explored under the optical reaction conditions, and the experimental outcomes are listed in Table 6. For symmetric and asymmetric secondary amines (Table 6, entries 1–4), high conversion rates and selectivity were achieved and a small amount of benzaldehyde was detected. For 1,2,3,4-tetrahydroquinoline, high selectivity (99%) for quinoline was obtained by extending the reaction time (Table 6, entry 5). For 1,2,3,4-tetrahydroisoquinoline, one molecule of hydrogen was removed in the reaction, and the conversion rate reached 91.2% within 2 h (Table 6, entry 6). However, the further oxidation of 3,4-dihydroisoquinoline to isoquinoline during the reaction reduced the selectivity.

**Figure 8:**
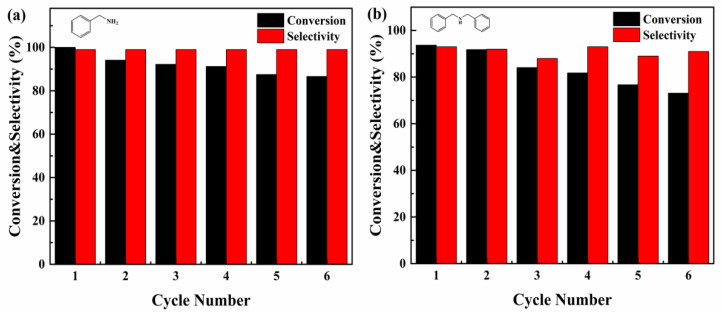
Photocatalytic cycle of benzylamine coupling reaction (**a**); photocatalytic cycle of oxidative dehydrogenation of dibenzylamine (**b**).

In order to study the stability of the PCN/BiOCl-0 photocatalyst, cyclic experiments were carried out. Figure 8 shows the cyclic reaction tests carried out using benzylamine (Figure 8a) and dibenzylamine (Figure 8b) as substrates. The selectivity and conversion rate remained stable after five cycles, and a slight decrease was observed. The used catalyst in Figure 8b was collected, and the stability of the catalysts was investigated using XRD, UV-Vis DRS, FT-IR and XPS. Figure S4a–c are the XRD, UV-Vis DRS and FT-IR spectra; Figure S5 contains the XPS spectra of before and after PCN/BiOCl-0, respectively. The positions of the main characteristic peaks had not changed after the reaction, indicating that the structures of catalysts were stable after the reaction. The catalytic activity of the photocatalyst was decreased, possibly due to the mechanical damage during the cyclic process. Overall, the PCN/BiOCl-0 photocatalyst shows excellent chemical stability, making it a viable candidate for recycling.

### 3.5. Mechanism Analysis

**Figure 9:**
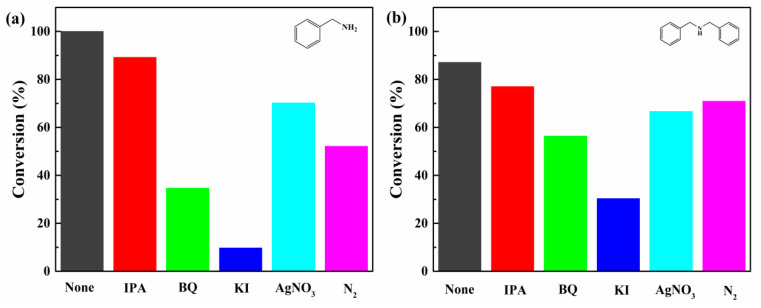
Radical trapping experiment of photocatalytic benzylamine coupling reaction (**a**) and radical trapping experiment of photocatalytic dibenzylamine dehydrogenation reaction (**b**).

To further investigate the main active species in the reaction, isopropanol (IPA), silver nitrate (AgNO_3_), p-benzoquinone (BQ) and potassium iodide (KI) were added to the PCN/BiOCl-0 photocatalytic amine oxidation reaction as ·OH, e^−^, ·O_2_^−^ and h^+^ scavengers, respectively (Figure 9). In the light of these experimental results, it may be proposed that ·OH has a minimal impact on the reaction, based on the slight reduction in conversion rate observed upon IPA addition. The addition of BQ and AgNO_3_ leads to a decrease in the conversion rates of benzylamine and dibenzylamine, indicating a certain involvement of ·O_2_^−^ and e^−^ in the reaction process. The conversion rates of benzylamine and dibenzylamine were decreased significantly after the addition of KI, demonstrating the primary involvement of h^+^ in the reaction process. In addition, the yield of product decreased significantly when the reaction was carried out under N_2_ atmosphere, further indicating the minor role of O_2_^−^ in the catalytic process.

According to characterization data obtained from UV-Vis diffuse reflectance spectroscopy and the Kubelka–Munk equation, the band gap values (E_g_) of BiOCl and PCN were found to be 3.60 eV and 2.80 eV, respectively. The potential energy values of the valence band (VB) and conduction band (CB) of the photocatalysts were determined using Equations (1) and (2):

E_CB_ = E_VB_ − E_g_ (1)

E_VB_ = χ − E_e_ + 0.5E_g_ (2)

**Figure 10:**
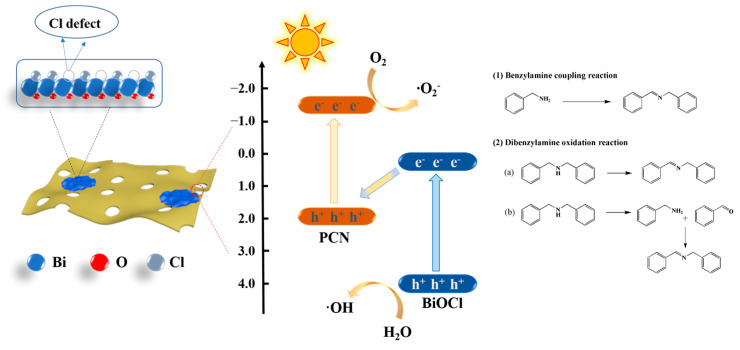
Mechanism of amine oxidation catalyzed using PCN/BiOCl-0 composite photocatalyst.

Herein, χ is used to denote the absolute electronegativity value of the sample, while E_e_ refers to the energy level of the free electrons relative to hydrogen (~4.5 eV) [33]. Based on the characterization results, a possible Z-scheme electron transfer mechanism is formulated and displayed in Figure 10. Under the light illumination, PCN and BiOCl produce photogenerated h^+^ and e^−^, respectively. The photogenerated h^+^ on the VB of PCN and e^−^ on the CB of BiOCl were recombined, while the photogenerated e^−^ on the CB of PCN reacted with O_2_ to form ·O_2_^−^. The generated h^+^ and ·O_2_^−^ play a crucial role in the amine oxidation reaction. In the reaction mechanism of benzylamine oxidation, benzylamine forms a free radical cation by reacting with photoinduced h^+^ captured at the VB of PCN; the benzylamine then reacts with ·O_2_^−^ to produce imine and hydrogen peroxide in the further reaction. The benzyl imine produced in the process undergoes a nucleophilic addition reaction with benzylamine to form N-phenylethylene-1-phenylmethylamine [33]. For the reaction mechanism of diphenylamine, there is a possible reaction pathway: diphenylamine undergoes direct oxidation and dehydrogenation to produce N-benzylidene-1-phenylmethanamine.

## 4. Conclusions

A high-specific-surface-area Z-heterojunction photocatalytic composite consisting of PCN and BiOCl-0 has been synthesized via the solvothermal method. The concentration of chlorine defects in the material can be adjusted by changing the solvent. The as-synthesized PCN/BiOCl-0 material was applied to the photocatalytic oxidation of amines and showed a remarkable improvement in photocatalytic activity, compared to pure PCN and BiOCl. Optical and S_BET_ analyses of the samples indicated that the upgraded photocatalytic activity of the PCN/BiOCl-0 material was mainly attributed to three factors: (1) more active sites exposed due to the increased specific surface area; (2) the presence of chlorine defects, which enhance light absorption ability and improve photocatalytic activity; (3) the Z-type electronic transfer mode, which improves the migration efficiency of photogenerated e^−^–h^+^ pairs. Cycling experiments confirmed that the PCN/BiOCl-0 Z-type heterojunction photocatalyst is a good recyclable photocatalyst.

## Figures and Tables

**Table 1:** Benzylamine coupling reaction catalyzed by different photocatalysts ^a^.

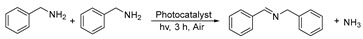			
**Entry**	**Catalyst**	**Conv. ^b^ (%)**	**Sel. ^b^ (%)**
1	PCN	40.1	99
2	BiOCl	54.5	99
3	PCN/Bi_2_O_3_	42.3	99
4	PCN/BiOCl-0	88.0	99
5	PCN/BiOCl-10	72.7	99
6	PCN/BiOCl-20	76.1	99

^a^ Reaction conditions: 0.01 g photocatalyst, 0.30 mmol benzylamine, 10 mL acetonitrile, 300 W xenon lamp irradiation for 3 h at room temperature. ^b^ The conversion rate and selectivity were obtained via GC.

**Table 2:** Optimum conditions for photocatalytic coupling reaction of benzylamine with PCN/BiOCl-0 ^a^.

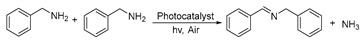				
**Entry**	**Time (h)**	**Catalyst Amount (g)**	**Conv. ^b^ (%)**	**Sel. ^b^ (%)**
1	3	0	2.7	99
2	3	0.01	49.6	99
3	3	0.02	68.3	99
4	3	0.03	66.2	99
5 ^c^	3	0.02	2.2	99
6	4	0.02	74.8	99
7	5	0.02	88.5	99
8	6	0.02	97.2	99

^a^ Reaction conditions: 0.50 mmol of benzylamine, 10 mL of acetonitrile, 300 W xenon lamp, room temperature. ^b^ Conversion and selectivity were measured via GC. ^c^ No light was used.

**Table 3:** Photocatalytic oxidation of amine derivatives using PCN/BiOCl-0 composite ^a^.

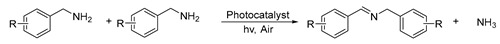				
**Entry**	**Product**	**Time (h)**	**Conv. ^b^ (%)**	**Sel. ^b^ (%)**
1	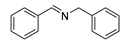	6	100	99
2	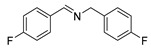	6	96.6	99
3	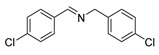	6	97.7	99
4	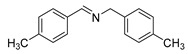	5	100	99
5	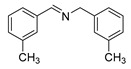	5/6	83.9/94.9	99
6	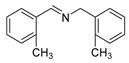	5/7	76.6/97.4	99
7	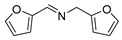	6	85.4	99
8	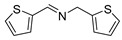	6	91	99
9	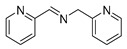	5	96.9	99

^a^ Reaction conditions: 0.02 g photocatalyst, 0.50 mmol substrate, 10 mL acetonitrile, 300 W xenon lamp, room temperature. ^b^ Conversion and selectivity were measured via GC.

**Table 4:** Comparative experiment on catalytic oxidation of dibenzylamine with different photocatalysts ^a^.

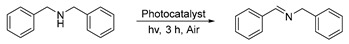			
**Entry**	**Catalyst**	**Conv. ^b^ (%)**	**Sel. ^b^ (%)**
1	PCN	25.7	99
2	BiOCl	36.0	99
3	PCN/Bi_2_O_3_	52.0	99
4	PCN/BiOCl-0	59.1	99
5	PCN/BiOCl-10	39.9	99
6	PCN/BiOCl-20	45.2	99

^a^ Reaction conditions: 0.01 g photocatalyst, 0.15 mmol dibenzylamine, 10 mL acetonitrile, 300 W xenon lamp irradiation for 3 h, room temperature. ^b^ The conversion rate and selectivity were obtained through GC.

**Table 5:** Optimum conditions for photocatalytic oxidation of dibenzylamine using PCN/BiOCl-0 ^a^.

Entry	Time (h)	Catalyst Amount (g)	Conv. ^b^ (%)	Sel. ^b^ (%)
1	3	0	5.8	99
2	3	0.01	40.5	99
3	3	0.02	53.5	99
4	3	0.03	59.3	98
5 ^c^	3	0.02	0	--
6	4	0.02	70.1	96
7	5	0.02	82.0	95
8	6	0.02	93.7	93
9	7	0.02	100	91

^a^ Reaction conditions: 0.50 mmol dibenzylamine, 10 mL acetonitrile, 300 W xenon lamp, room temperature. ^b^ Conversion rate and selectivity were measured via GC. ^c^ No light irradiation.

**Table 6:** Photocatalytic oxidation of amine derivatives using PCN/BiOCl-0 ^a^.

Entry	Substrate	Time (h)	Conv. ^b^ (%)	Product (sel. ^b^ (%))
1	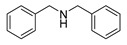	6	93.7	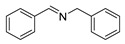 (93)
2	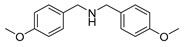	4	84.9	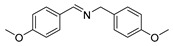 (91)
3	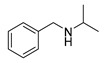	6	90.6	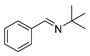 (99)
4	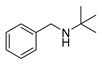	7	94.0	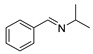 (90)
5	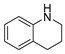	26	79.3	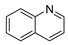 (99)
6	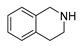	2	91.2	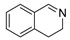 (91)

^a^ Reaction conditions: 0.02 g photocatalyst, 0.25 mmol substrate, 10 mL acetonitrile, 300 W xenon lamp, room temperature. ^b^ Conversion and selectivity were obtained through GC.

## Data Availability

The datasets generated during and/or analysed during the current study are available from the corresponding author on reasonable request.

## Supplementary Materials

The following supporting information can be downloaded at: https://www.mdpi.com/article/10.3390/polym15204145/s1. Table S1: Analysis of element content of Bi and Cl; Table S2: Surface area analysis of samples; Figure S1: O 1s spectra of PCN; Figure S2: UV-Vis DRS and band gap energy of PCN (a) and BiOCl (b); Figure S3: Photocurrent curve of samples (a), optical impedance (b) and dark impedance plot (c) of samples; Figure S4: The XRD patterns (a), UV-Vis DRS (b) and FT-IR patterns (c) of before and after PCN/BiOCl; Figure S5: XPS survey spectra of before and after PCN/BiOCl (a); XPS spectra of before and after PCN/BiOCl (Bi, C, N, O, Cl) (b–f).
