# Supplementary material for: PCN/BiOCl Polymer-Based Heterojunction with Rich Chlorine Defects for Photocatalytic Amine Oxidation
Source: Polymers (Basel). 2023 Oct 19;15(20):4145. doi: 10.3390/polym15204145 (PMC10611391; doi:10.3390/polym15204145)
Supplement: Supplementary file 1 [file polymers-15-04145-s001.zip › polymers-2614290-supplementary.pdf]

Supplementary Materials: PCN/BiOCl Polymer-Based Heterojunction with Rich Chlorine Defects for Photocatalytic Amine Oxidation

Guichuan Xu †, Zhuhan Wang †, Yefeng Chen, Li Qin and Limei Zhou *

Chemical Synthesis and Pollution Control Key Laboratory of Sichuan Province, China West Normal University, Nanchong 637002, China; xuguichuan2023@163.com (G.X.); 15760592910@163.com (Z.W.); 13890791350@163.com (Y.C.); cwnuqinli@163.com (L.Q.)

* Correspondence: cwnuzhoulimei@163.com; Tel.: +86-817-2568081

† These authors contributed equally to this work.

Table S1. Analysis of element content of Bi and Cl.

Catalyst	Bi (wt%)	Cl (wt%)	Bi: Cl (mol ratio)
BiOCl	81.46	11.78	1.17
PCN/BiOCl-0	3.70	0.11	5.71
PCN/BiOCl-10	5.46	0.43	2.16
PCN/BiOCl-20	7.32	0.94	1.32

Table S2. Surface area analysis of samples.

Catalyst	PCN	PCN/BiOCl-0	PCN/BiOCl-10	PCN/BiOCl-20
S_{BET} ($m^2 \cdot g^{-1}$)	30.04	65.86	58.97	39.67

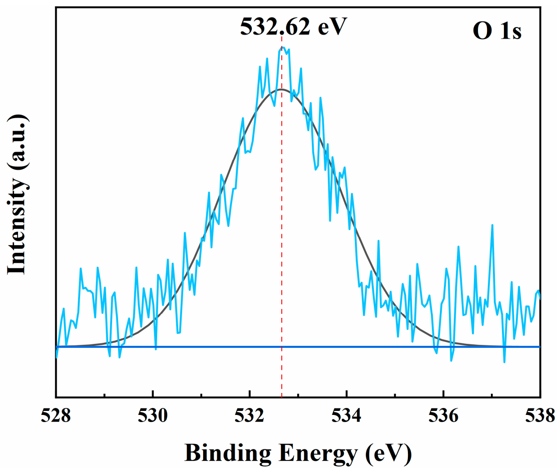

Figure S1. O 1s spectra of PCN.

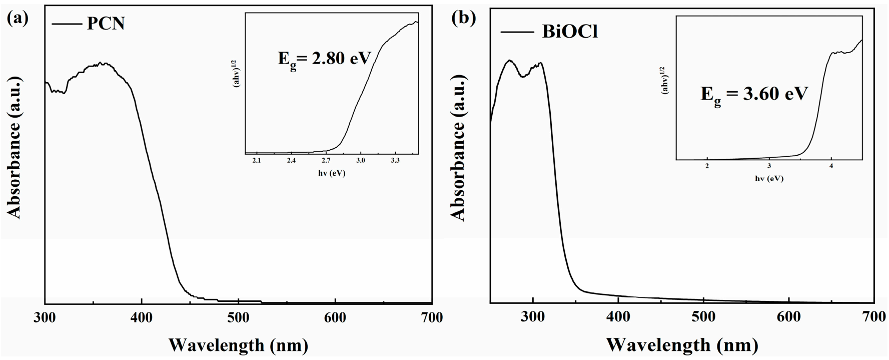

Figure S2. UV-Vis DRS and band gap energy of PCN (a) and BiOCl (b).

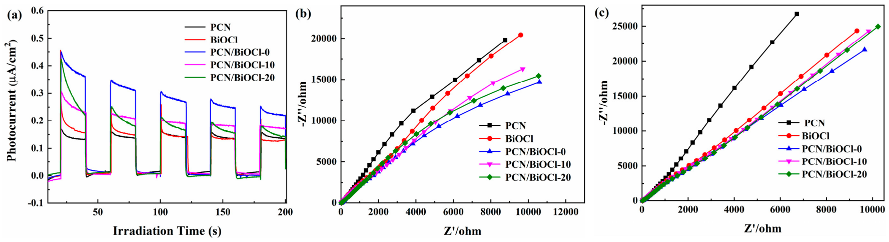

Figure S3. Photocurrent curve of samples (**a**), optical impedance (**b**) and dark impedance plot (**c**) of samples.

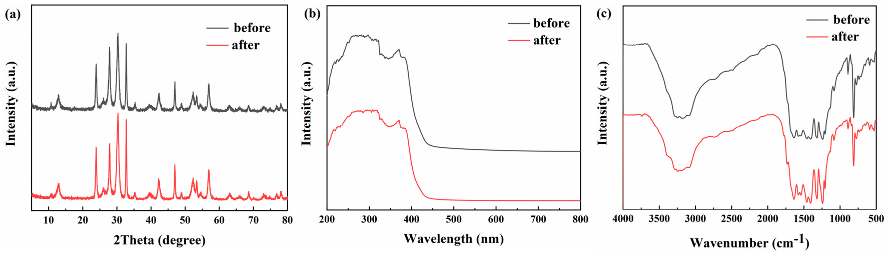

Figure S4. The XRD patterns (**a**), UV-Vis DRS (**b**) and FT-IR patterns (**c**) of before and after PCN/BiOCl.

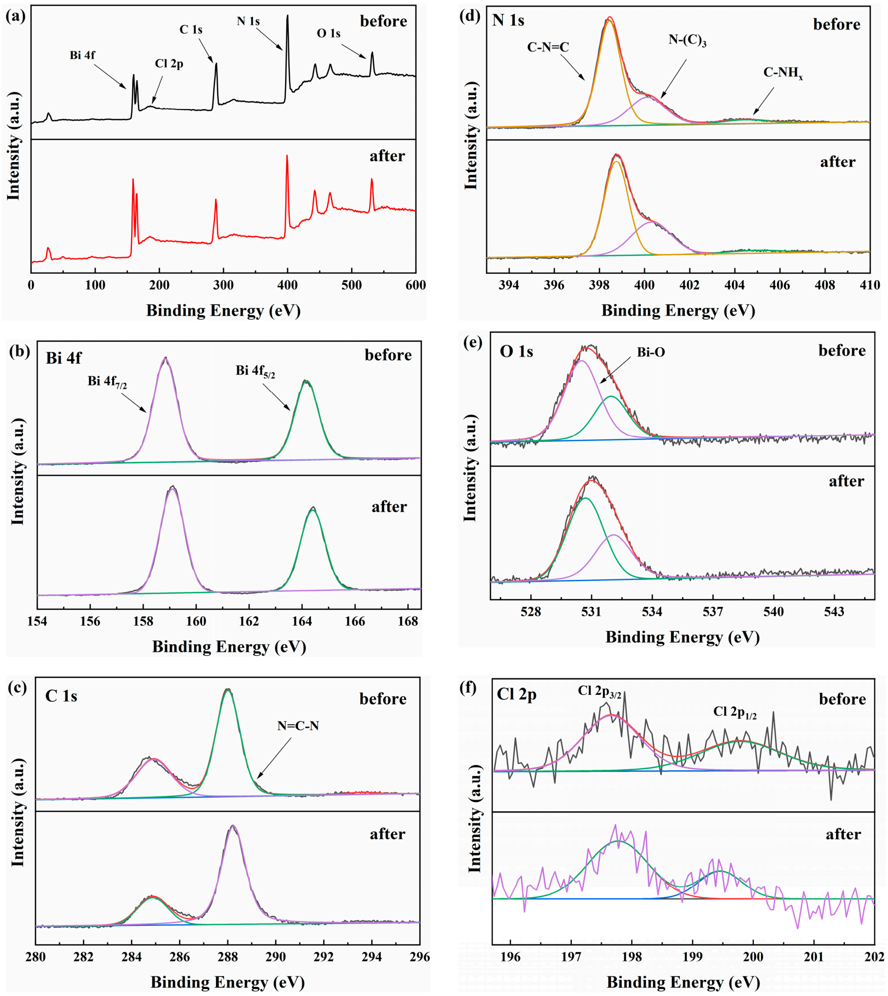

Figure S5. XPS survey spectra of before and after PCN/BiOCl (**a**); XPS spectra of before and after PCN/BiOCl (Bi, C, N, O, Cl) (**b–f**).
